# Specific EEG resting state biomarkers in FXS and ASD

**DOI:** 10.1186/s11689-024-09570-9

**Published:** 2024-09-09

**Authors:** Mélodie Proteau-Lemieux, Inga Sophia Knoth, Saeideh Davoudi, Charles-Olivier Martin, Anne-Marie Bélanger, Valérie Fontaine, Valérie Côté, Kristian Agbogba, Keely Vachon, Kerri Whitlock, Hazel Maridith Barlahan Biag, Angela John Thurman, Cory Rosenfelt, Flora Tassone, Julia Frei, Lucia Capano, Leonard Abbeduto, Sébastien Jacquemont, David Hessl, Randi Jenssen Hagerman, Andrea Schneider, Francois Bolduc, Evdokia Anagnostou, Sarah Lippe

**Affiliations:** 1 Department of Psychology, University of Montreal, Montreal, QC Canada; 2 Research Center of the Sainte-Justine University Hospital, Montreal, QC Canada; 3 Department of Neuroscience, University of Montreal, Montreal, QC Canada; 4 University of Alberta, Edmonton, AB Canada; 5 Department of Pediatrics and MIND Institute, University of California Davis School of Medicine, Sacramento, CA USA; 6 Department of Psychiatry and Behavioral Sciences and MIND Institute, University of California Davis School of Medicine, Sacramento, CA USA; 7 Department of Pediatric Neurology, University of Alberta, Edmonton, AB Canada; 8 Department of Biochemistry and Molecular Medicine, University of California Davis School of Medicine, Sacramento, CA USA; 9 McMaster University of Ottawa, Ottawa, ON Canada; 10 Queen’s University of Kingston, Kingston, ON Canada; 11 Department of Pediatrics, University of Montreal, Montreal, QC Canada; 12 Department of Pediatrics, University of Alberta, Edmonton, AB Canada; 13 Department of Paediatrics, University of Toronto, Toronto, ON Canada; 14 Holland Bloorview Research Center, Toronto, ON Canada

**Keywords:** Fragile X syndrome, Autism spectrum disorder, Resting state EEG, Signal complexity, Multi scale entropy, Alpha peak frequency, Power spectral density, Neurodevelopment, Cognition

## Abstract

**Background:**

Fragile X syndrome (FXS) and autism spectrum disorder (ASD) are neurodevelopmental conditions that often have a substantial impact on daily functioning and quality of life. FXS is the most common cause of inherited intellectual disability (ID) and the most common monogenetic cause of ASD. Previous literature has shown that electrophysiological activity measured by electroencephalogram (EEG) during resting state is perturbated in FXS and ASD. However, whether electrophysiological profiles of participants with FXS and ASD are similar remains unclear. The aim of this study was to compare EEG alterations found in these two clinical populations presenting varying degrees of cognitive and behavioral impairments.

**Methods:**

Resting state EEG signal complexity, alpha peak frequency (APF) and power spectral density (PSD) were compared between 47 participants with FXS (aged between 5–20), 49 participants with ASD (aged between 6–17), and 52 neurotypical (NT) controls with a similar age distribution using MANCOVAs with age as covariate when appropriate. MANCOVAs controlling for age, when appropriate, and nonverbal intelligence quotient (NVIQ) score were subsequently performed to determine the impact of cognitive functioning on EEG alterations.

**Results:**

Our results showed that FXS participants manifested decreased signal complexity and APF compared to ASD participants and NT controls, as well as altered power in the theta, alpha and low gamma frequency bands. ASD participants showed exaggerated beta power compared to FXS participants and NT controls, as well as enhanced low and high gamma power compared to NT controls. However, ASD participants did not manifest altered signal complexity or APF. Furthermore, when controlling for NVIQ, results of decreased complexity in higher scales and lower APF in FXS participants compared to NT controls and ASD participants were not replicated.

**Conclusions:**

These findings suggest that signal complexity and APF might reflect cognitive functioning, while altered power in the low gamma frequency band might be associated with neurodevelopmental conditions, particularly FXS and ASD.

## Background

Fragile X syndrome (FXS) and autism spectrum disorder (ASD) are two neurodevelopmental conditions that often impair daily functioning and quality of life. Approximately 0.02% of individuals have FXS [1], while ASD affects 1% of the global population [2]. More males with FXS and ASD are affected than females, with male:female ratios of around 2:1 for FXS and 3:1 for ASD [3]. FXS is a genetic disorder caused by a cytosine-guanine-guanine (CGG) tri-nucleotide expansion in the *Fragile X Messenger Ribonucleoprotein gene 1* (*FMR1*) situated on the bottom end of the X chromosome, leading to the loss of its expressed protein, FMRP. FMRP is essential to synaptic plasticity and maturation, and its absence is associated with excessive synthesis of proteins involved in neuronal development [4]. Although the etiology of FXS is well known, the exact causes of ASD are more complex. An interaction between genetic mutations and environmental factors, including prenatal risk factors and environmental exposures during critical neurodevelopmental periods, is thought to contribute to ASD [5]. Though the symptomatology of participants with FXS and ASD is widely heterogeneous, there is a high comorbidity between the two disorders, with FXS being the most common monogenetic cause of ASD [6, 7]. Specifically, 90% of males with FXS manifest symptoms of autism, while approximately 60% have an official diagnosis of ASD [8, 9]. Literature regarding females with FXS is not as clear-cut. However, it is estimated that around 20% of them meet the criteria for ASD [9]. Language deficits, repetitive behaviors, and impaired social skills are the most observed symptoms of ASD in FXS. Importantly, individuals with FXS manifesting symptoms of ASD also present a more severe clinical phenotype [10] than individuals with a single diagnosis of either FXS or ASD. Both FXS and ASD are associated with cognitive and behavioral impairments, such as intellectual disability (ID), attention deficit hyperactivity disorder (ADHD), and irritability. The prevalence of ID in FXS is around 85% in males and 30% in females [11, 12], while 70% of individuals with ASD present some level of ID [13]. Lastly, females with FXS typically manifest less clinical symptoms than males given that they carry an unaffected X chromosome.

In addition to clinical symptoms, severe neurophysiological alterations are present in both conditions. However, their degree of overlap is unclear. An imbalance between excitatory and inhibitory cortical mechanisms is thought to contribute to cortical hyperexcitability, a hallmark of FXS [14]. Rubenstein and Merzenich [15] proposed a neurobiological model of E/I imbalances and hypothesized that overexcitation of neural circuits could be explained by increased glutamatergic signaling or decreased GABAergic signalling in the brain of individuals with ASD. Bear and colleagues [16] introduced the metabotropic glutamate receptors (mGluRs) theory to explain E/I imbalances in FXS. Taken together, these E/I models suggest that overactivation of mGluRs and reduced activation of the GABAergic system lead to excessive neuronal excitability and hypoinhibition [17] in individuals with FXS and ASD. Previous literature has shown that electrophysiological responses, as measured with electroencephalogram (EEG) during resting state, are perturbated in FXS and ASD [18]. In both conditions, participants showed, relative to neurotypical (NT) controls, lower power in the alpha frequency band [19–21], the prominent oscillations in human adults during resting state, as well as increased gamma power [20, 22]. Furthermore, alpha peak frequency (APF), a marker sensitive to brain maturation, has been found to be diminished in many neurodevelopmental conditions, including FXS and ASD [20, 23]. Signal complexity was shown to be an important biomarker for brain maturation and atypical development as it has been found to be lower in many neurodevelopmental disorders, including ADHD and Tourette syndrome as well as FXS and ASD [20, 21, 24–29]. Specifically, signal complexity in coarser grained scales is lower in participants with FXS, which was associated with brain maturation alterations [20].

Several EEG biomarkers have been found to be altered in FXS and ASD. However, it has yet to be determined whether FXS and ASD share patterns of alterations, or if they display distinct profiles. In this study, we investigated how age affected the EEG biomarkers in each group, and how each group showed distinctive electrophysiological profiles on signal complexity and power spectral density (PSD). Finally, we explored the impact of cognitive functioning as measured by intellectual quotient (IQ) on EEG alterations. We first hypothesized that NT controls would show typical maturational changes, but that these typical age effects would not be observed in participants with FXS and ASD. We also hypothesized that participants with FXS and ASD would present reduced signal complexity compared to NT controls, and that participants with FXS and ASD would manifest lower APF and alpha power, as well as exaggerated beta, low gamma, and high gamma power compared to NT controls. The samples selected for this study presented more severe cognitive and behavioral impairments in participants with FXS compared to a group of participants with ASD who were mostly high functional. Further, more than 50% of participants with FXS also had a diagnosis of ASD. Therefore, we hypothesized that participants with FXS would also manifest reduced signal complexity, APF and alpha power, as well as increased gamma power compared to participants with ASD. To our knowledge, this study is the first to directly compare the EEG profiles of participants with FXS and ASD.

## Methods

### Participants

Forty-seven participants with a confirmed genetic diagnosis of FXS, and 49 participants with a clinical diagnosis of ASD were recruited for this study. The genetic diagnosis was based on genetic screening and FXS was diagnosed when > 200 repetitions of CGG were present. Twenty-four participants with FXS had a comorbid diagnosis of ASD, and 34 met the criteria for ID, with a nonverbal intelligence quotient (NVIQ) < 70, as measured with a cognitive assessment. Participants of the ASD group were evaluated by a clinician with expertise for the present project, and the diagnosis was based on meeting the criteria of the Diagnostic and Statistical Manual of Mental Disorders, Fifth Edition (DSM-5) for ASD. The Autism Diagnostic Observation Schedule, Second Edition (ADOS-2) was administered by the same clinician to support the diagnosis. ASD participants were not diagnosed with any other genetic conditions, but five of them also met the criteria for ID (NVIQ < 70). Individuals with ASD were recruited via a multicentric clinical trial and one of the admissibility criteria included having complex language to qualify for ADOS-2 modules 3 or 4. In the FXS cohort, no such level of language was required. Participants with FXS and ASD who were taking medication were allowed to keep doing so for the duration of the study. Fifty-two NT controls with a similar age and gender distribution were also recruited. The admissibility criteria for NT controls included participants with no neurological or psychological conditions, and participants not taking any medication. Table 1 provides the demographics of the study population, Table 2 presents the differences in age and NVIQ between the three groups, and Table 3 describes the clinical populations, FXS and ASD, in detail. At least 1 min of artifact-free resting state EEG recording was available for all participants included in the analyses.

**Table 1:** Demographics of the study population

	**FXS**	**ASD**	**Controls**
**N**	47	49	52
Males (n, %)	35 (74.5%)	36 (73.5%)	26 (50%)
Females (n, %)	12 (25.5%)	13 (26.5%)	26 (50%)
**Age**			
Mean ± SD	12.79 ± 4.47	12.80 ± 3.30	10.44 ± 3.97
Range	5–20	6–17	5–20
**NVIQ**			
Mean ± SD	62.28 ± 22.52	95.90 ± 18.27	110.54 ± 10.39
Range	31–123	46–138	77–125

**Table 2:** Differences in age and NVIQ between the groups. Asterisks represent statistically significant differences at the 5% level

	**FXS vs NT controls**	**ASD vs NT controls**	**FXS vs ASD**
**Age**			
ANOVA	*F*(1,97) = 7.64 *p* = .007*	*F*(1,99) = 10.43 *p* = .002*	*F*(1,94) = .00 *p* = .99
**NVIQ**			
ANOVA	*F*(1,97) = 235.62 *p* < .001*	*F*(1,99) = 24.87 *p* < .001*	*F*(1,94) = 74.57 *p* < .001*

**Table 3:** Comorbid diagnoses and medication in the clinical populations

	**FXS**	**ASD**
**N**	47	49
**Comorbid diagnoses**		
ASD (%)	24 (51.06%)	NA
ID (%)	34 (72.34%)	5 (10.20%)
Epilepsy (%)	4 (8.51%)	1 (2.04%)
ADHD (%)	19 (40.43%)	25 (51.02%)
**Medication**		
Antipsychotics (%)	6 (12.77%)	4 (8.16%)
Antidepressants (%)	13 (27.66%)	13 (26.53%)
Anxiolytics (%)	1 (2.13%)	1 (2.04%)
Psychostimulants (%)	21 (44.68%)	21 (42.86%)
Anticonvulsants (%)	0 (0%)	1 (2.04%)
Cannabidiol (%)	3 (6.38%)	0 (0%)

FXS participants were recruited via the genetic clinics of the CHU Sainte-Justine in Montreal, the University of Alberta, and the University of California Davis MIND Institute. ASD participants were recruited during a multicentric placebo-controlled randomized clinical trial conducted at the University of Toronto Holland Bloorview Kids Rehabilitation Hospital, the McMaster University, and Queen’s University. Baseline data from the trial were used for the analyses. Recordings for all NT controls were accessed through the NED laboratory database at the CHU Sainte-Justine. The studies’ protocols were approved by the ethics committees of all involved centres and written informed consent was obtained from all participants or legal caregivers.

### Cognitive and behavioral measures

To assess the NVIQ of FXS participants, the Leiter-R or Leiter-3 brief IQ [30] were used. For ASD participants, the cognitive assessment was carried out using the Standford-Binet Intelligence Scales, Fifth Edition (SB-V [31],) or the Wechsler Abbreviated Scale of Intelligence, Second Edition (WASI-II [32],). To quantify behavioral problems, the revised version of the Aberrant Behavior Checklist for Community (ABC-C-FX), specifically developed for the FXS population [33], was used. The ABC-C-FX is a 58-item questionnaire that includes 6 subscales: irritability, hyperactivity, lethargy, stereotypic behavior, inappropriate speech, as well as social withdrawal, which is highly common in FXS and ASD. Higher scores are associated with more severe aberrant behavior. To measure adaptative behavior, the Vineland Adaptative Behavior Scales, Third Edition (Vineland-III), was used. The Vineland-III is the most commonly used instrument to evaluate adaptative behavior and daily functioning in individuals with neurodevelopmental disorders [34]. Higher scores reflect better adaptative functioning. Table 4 shows the cognitive and behavioral differences between the clinical populations.

**Table 4:** Cognitive and behavioral differences between FXS and ASD participants. Asterisks represent statistically significant differences at the 5% level

	**FXS**	**ASD**
**NVIQ**		
Mean ± SD	62.28 ± 22.52	95.90 ± 18.27
ANOVA	*F*(1,94) = 74.57, *p* < .001*	
**ABC-C-FX scores**		
Composite score (mean ± SD)	51.84 ± 36.28	32.14 ± 22.51
ANOVA	*F*(1,91) = 10.12, *p* = .002*	
Irritability subscale (mean ± SD)	16.09 ± 15.20	9.65 ± 10.50
ANOVA	*F*(1,93) = 5.82, *p* = .018*	
Lethargy subscale (mean ± SD)	8.44 ± 7.18	4.88 ± 3.84
ANOVA	*F*(1,92) = 9.22, *p* = .003*	
Stereotypy subscale (mean ± SD)	5.96 ± 5.40	3.20 ± 2.94
ANOVA	*F*(1,94) = 9.74, *p* = .002*	
Hyperactivity subscale (mean ± SD)	11.48 ± 7.75	7.84 ± 6.80
ANOVA	*F*(1,93) = 5.95, *p* = .017*	
Inappropriate speech subscale (mean ± SD)	5.26 ± 3.64	3.06 ± 2.95
ANOVA	*F*(1,93) = 10.53, *p* = .002*	
Social withdrawal (mean ± SD)	3.66 ± 3.19	3.51 ± 3.39
ANOVA	*F*(1,94) = .05, *p* = .825	
**Vineland-III scores**		
Adaptative Behavior Composite score (mean ± SD)	51.29 ± 23.22	71.47 ± 11.59
ANOVA	*F*(1,78) = 26.66, *p* < .001*	
Communication domain (mean ± SD)	43.68 ± 21.49	71.08 ± 15.36
ANOVA	*F*(1,78) = 44.16, *p* < .001*	
Daily Living Skills domain (mean ± SD)	58.74 ± 29.31	75.39 ± 13.17
ANOVA	*F*(1,78) = 12.03, *p* < .001*	
Socialization domain (mean ± SD)	54.48 ± 23.90	72.02 ± 15.80
ANOVA	*F*(1,78) = 15.64, *p* < .001*	

### Procedure

Participants were prepared for the EEG sessions using pictograms and videos. During net installation, a movie was presented to increase compliance from participants. For the resting state recording, participants were asked to move as little as possible while staying relaxed and to keep their eyes on the screen, where a movie was shown. Resting state was recorded as the first task of the protocol, and the recording went on until a total of four (non-consecutive) minutes of movement-free signal were obtained. Recording sessions lasted on average seven minutes, regardless of group adherence. Recordings were carried out in dedicated EEG suites at the different sites. Three different EEG systems were used: 1) an EGI 128-electrode dense array system (Magstim EGI, Eugene, OR, USA) was used for all NT controls, FXS participants from CHU Sainte-Justine and University of Alberta, as well as for ASD participants from Queens’ University; 2) a BrainVision 64-electrode Acticap (Brain Products, Germany) was used for ASD participants from University of Toronto Holland Bloorview Kids Rehabilitation Hospital, and McMaster University; and 3) a BrainVision 32-electrode Acticap (Brain Products, Germany) was used for FXS participants from MIND Institute. For the EGI system, the Cz electrode was used as online reference, and an isolated common sensor was located between CPz and Pz. For Brain Products, FCz electrode was used as online reference and AFz was used as ground. EEG data were digitized and processed at a sampling rate of 1000 Hz with a bandpass filter of 0.1-500 Hz. Impedances were kept below 40kΩ for the EGI system, and below 10kΩ for the BrainVision systems.

### EEG signal processing

#### Pre-processing

**Fig. 1:**
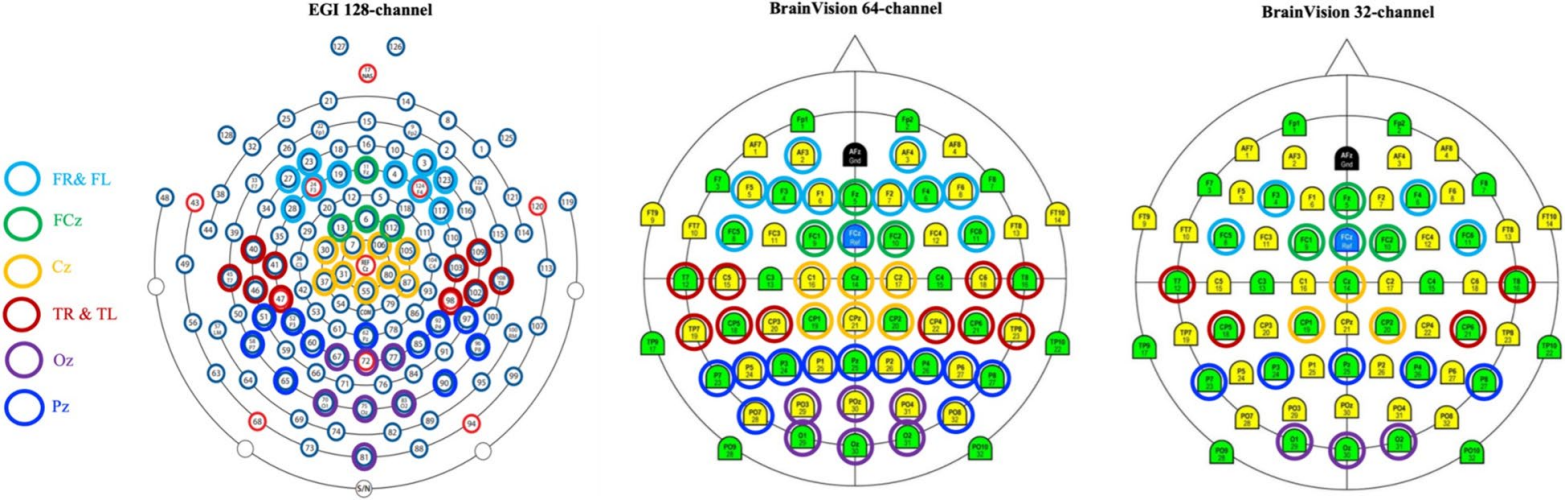
Comparative map of each EEG system

Pre-processing analyses were carried out using MATLAB (version R2020b) and the EEGLAB toolbox (version 14.1.2). The first pre-processing step consisted of applying a FIR bandpass filter with filter order 6600 using a Hamming window. The low cutoff was set to 0.5 Hz, the high cutoff was set to 150 Hz, a 0.5 Hz transition band was used and the maximum passband ripple was 0.0022 dB. A 60 Hz notch filter was applied. Then, external channels were automatically removed due to low quality signal around the face and neck. For the EGI 128-channel cap, 28 channels were removed, and for the 32- and 64-channel caps, 6 channels were removed. A semi-automatic procedure was used to remove the remaining noisy electrodes: Firstly, electrodes with a total standard deviation of > 120 μV and < 2 μV were automatically removed, and secondly, remaining noisy electrodes were manually removed during visual inspection. For the next step, data were re-referenced to the average reference. Then, independent component analysis (ICA) was carried out, and components reflecting blinks, saccades, and cardiac activity were manually removed. Continuous data were segmented into 2 s epochs. Finally, artifact rejection was carried out using a semi-automatic procedure. Epochs with amplitudes > 200 μV and < -200 μV were automatically removed, and then epochs containing any kind of remaining artifacts were manually removed during visual inspection. In total, eight regions of interest (ROIs) were defined for the analyses: central (Cz), frontal right (FR), frontal left (FL), fronto-central (FCz), occipito-central (Oz), parieto-central (Pz), temporal right (TR), and temporal left (TL). The electrodes contained in each ROI were chosen to match between the three different EEG systems and are presented in Table 5. Figure 1 shows the comparative map of each system.

**Table 5:** Electrodes chosen for each ROI in all three EEG systems

	**EGI 128-channel**	**BrainVision 64-channel**	**BrainVision 32-channel**
Cz	'E7', 'E30', 'E31', 'E37', 'E55', 'E80', 'E87', 'E105', 'E106'	'C1', 'C2', 'Cz', 'CP1', 'CP2', 'CPz'	'Cz', 'CP1', 'CP2'
FR	'E3', 'E4', 'E117', 'E123', 'E124'	'AF4', 'F6', 'F4', 'F2', 'FC6'	'F4', 'FC6'
FL	'E19', 'E23', 'E24', 'E27', 'E28'	'AF3', 'F5', 'F3', 'F1', 'FC5'	'F3', 'FC5'
FCz	'E6', 'E11', 'E13', 'E112'	'Fz', 'FC1', 'FC2', 'FCz'	'Fz', 'FC1', 'FC2'
Oz	'E67', 'E70', 'E72', 'E75', 'E77', 'E81', 'E83'	'POz', 'PO3', 'PO4', 'O1', 'Oz', 'O2'	'O1', 'Oz', 'O2'
Pz	'E51', 'E52', 'E58', 'E60', 'E62', 'E65', 'E85', 'E90', 'E92', 'E96', 'E97'	'Pz', 'P1', 'P2', 'P7', 'P3', 'P4', 'P8', 'P5', 'P6', 'PO7', 'PO8'	'P7', 'P3', 'P4', 'P8', 'Pz'
TR	'E98', 'E102', 'E103', 'E108', 'E109'	'T8', 'C6', 'TP8', 'CP6', 'CP4'	'C4', 'CP6'
TL	'E40', 'E41', 'E45', 'E46', 'E47'	'T7', 'C5', 'TP7', 'CP5', 'CP3'	'C3', 'CP5'

#### Normalization

To allow comparison between the different EEG systems, a normalization procedure was carried out individually for each participant using z-scores [35]. Specifically, the mean of the signal was subtracted from the original signal, and then divided by the standard deviation of the signal. Afterwards, channel interpolation was performed to avoid data loss following channel rejection.

#### Signal complexity

A multiscale entropy (MSE) algorithm was used to measure signal complexity at rest. This algorithm generates multiple timescales by down-sampling the original EEG signal using a coarse-graining procedure [36]. In the present study, coarse-grained series up to scale 40 were produced for each ROI, where timescale 1 is the original series, timescale 2 is the average of two consecutive values from the original series, timescale 3 is the average of three values from the original series, and so forth [24]. Here, the parameters used to calculate MSE were *m* = 2 and *r* = 0.5, which indicates that 50% of the original series standard deviation were considered in the calculation of the MSE. For the analyses, two MSE components were analysed: 1) lower complexity scales = average of timescales from 1–20; and 2) higher complexity scales = average of timescales from 21–40.

#### PSD

A fast Fourier transform (FFT) algorithm was used to compute power spectral density in terms of power per frequency for all frequencies from 1 to 100 Hz, with a resolution of 0.5 Hz. Six frequency bands were used for the analyses: 1) delta = 1-3 Hz; 2) theta = 4-7 Hz; 3) alpha = 8-12 Hz; 4) beta = 13-30 Hz; 5) low gamma = 30-50 Hz; and 6) high gamma = 70-100 Hz [37]. Frequencies from 50-70 Hz were not considered due to the applied notch filter at 60 Hz. APF was defined as the frequency with the maximum amplitude between 4.5 and 12 Hz. Frequencies from 4.5 to 7 Hz were included since APF is generally situated in the theta range in FXS participants.

### Statistical analyses

All statistical analyses were performed with SPSS Statistics version 27 (IMB Corp., Armonk, NY, USA). Normality of data was confirmed with z-scores and skewness/kurtosis criteria, where values between -1 and 1 were accepted. The significance level (α) was set to 5% (*p* < 0.05). First, Pearson correlation analyses were performed to investigate associations between all EEG markers and age in the three groups. Since EEG markers correlated between all the ROIs, average correlations between outcome variables were considered when correcting significance levels for multiple correlations using Bonferroni’s method to avoid overcorrection [38]. For EEG markers that significantly correlated with age, we conducted multivariate analysis of covariance (MANCOVA) with age as covariate to compare EEG markers between the three groups, with follow-up ANOVAs using Bonferroni corrections to investigate significant interactions. We used Wilks’ lambda to compare groups in MANCOVA results. Given that we wanted to explore the impact of cognitive functioning on EEG markers, MANCOVAs with age, when appropriate, and NVIQ score as covariates were subsequently performed to determine the effect of cognitive functioning on EEG alterations, and ANOVAs with Bonferroni corrections were used for significant interactions. Lastly, as the number of electrodes varied among systems, differences between groups were considered substantial when at least three ROIs displayed statistically significant results, representing a minimum of 25% of scalp coverage.

## Results

### Developmental effects

#### Lower complexity scales and age

Significance level for correlations was Bonferroni-corrected to *p* < 0.017, accounting for the average correlation between the eight ROIs [38]. In FXS participants, positive correlations between lower complexity scales and age were found in Cz (*r* = 0.36, *p* = 0.015), FCz (*r* = 0.39, *p* = 0.007), Oz (*r* = 0.41, *p* = 0.004), Pz (*r* = 0.43, *p* = 0.003), TL (*r* = 0.37, *p* = 0.012), and TR (*r* = 0.37, *p* = 0.012). In ASD participants, positive correlations were found across almost all ROIs (Cz: *r* = 0.49, *p* < 0.001; FL: *r* = 0.37, *p* = 0.010; FCz: *r* = 0.50, *p* < 0.001; Oz: *r* = 0.56, *p* < 0.001; Pz: *r* = 0.57, *p* < 0.001; TL: *r* = 0.52, *p* < 0.001; TR: *r* = 0.53, *p* < 0.001) except FR (*r* = 0.34, *p* = 0.019), where the correlation did not survive correction. In NT controls, lower complexity scales positively correlated with age in all ROIs (Cz: *r* = 0.52, *p* < 0.001; FL: *r* = 0.41, *p* = 0.004; FR: *r* = 0.43, *p* = 0.002; FCz: *r* = 0.53, *p* < 0.001; Oz: *r* = 0.57, *p* < 0.001; Pz: *r* = 0.59, *p* < 0.001; TL: *r* = 0.53, *p* < 0.001; TR: *r* = 0.54, *p* < 0.001).

#### Higher complexity scales and age

Significance level for correlations was corrected to *p* < 0.007. In FXS participants, negative correlations were found in FL (*r* = -0.31; *p* = 0.036) and FR (*r* = -0.31; *p* = 0.04), but they did not survive correction for multiple correlations. In ASD participants and NT controls, no correlations were found.

#### APF and age

Significance level for correlations was corrected to *p* < 0.017. In FXS participants, APF did not correlate with age in any ROI. In ASD participants, positive correlations were found in FCz (*r* = 0.37; *p* = 0.010), and TR (*r* = 0.38; *p* < 0.008). Positive correlations were also found in Cz (*r* = 0.33, *p* = 0.022), FL (*r* = 0.31, *p* = 0.035), FR (*r* = 0.32, *p* = 0.027), Pz (*r* = 0.34, *p* = 0.02), and TL (*r* = 0.33, *p* = 0.021), but they did not survive correction. Lastly, in NT controls, positive correlations with age were found in all ROIs (Cz: *r* = 0.44, *p* = 0.001; FL: *r* = 0.53, *p* < 0.001; FR: *r* = 0.44, *p* = 0.001; FCz: *r* = 0.45, *p* = 0.001; Oz: *r* = 0.37, *p* = 0.007; Pz: *r* = 0.48, *p* < 0.001; TL: *r* = 0.50, *p* < 0.001; TR: *r* = 0.46, *p* < 0.001).

#### *Delta* and age

Significance level for correlations was corrected to *p* < 0.016. In FXS participants, negative correlations with age were found across almost all ROIs (Cz: *r* = -0.50, *p* < 0.001; FL: *r* = -0.60, *p* < 0.001; FCz: *r* = -0.55, *p* < 0.001; Oz: *r* = -0.49, *p* < 0.001; Pz: *r* = -0.46, *p* = 0.001; TL: *r* = -0.49, *p* < 0.001; TR: *r* = -0.50, *p* < 0.001) except FR (*r* = -0.34; *p* = 0.022), where the correlation did not survive correction. In ASD participants, delta correlated negatively with age in Cz (*r* = -0.46, *p* = 0.001), FCz (*r* = -0.40, *p* = 0.005), Oz (*r* = -0.42, *p* = 0.003), TL (*r* = -0.58; *p* < 0.009), and TR (*r* = -0.38, *p* = 0.008). Negative correlations were also found in FR (*r* = -0.34; *p* = 0.017) and Pz (*r* = -0.30; *p* = 0.036), but they did not survive correction for multiple correlations. In NT controls, negative correlations with age were found in almost all ROIs (Cz: *r* = -0.48, *p* < 0.001; FR: *r* = -0.40, *p* = 0.004; FCz: *r* = -0.55, *p* < 0.001; Oz: *r* = -0.34, *p* = 0.014; Pz: *r* = -0.47, *p* < 0.001; TL: *r* = -0.59, *p* < 0.001; TR: *r* = -0.38, *p* = 0.015) except FL (*r* = -0.18; *p* = 0.21).

#### *Theta* and age

Significance level for correlations was corrected to *p* < 0.009. In FXS participants, a negative correlation with age was found in FL (*r* = -0.41; *p* = 0.004). Negative correlations were also found in Cz (*r* = -0.36; *p* = 0.017) and Oz (*r* = -0.36; *p* = 0.014), but they did not survive correction. In ASD participants, theta did not correlate with age in any ROI. In NT controls, theta correlated negatively with age in FR (*r* = -0.44; *p* = 0.001), and Pz (*r* = -0.43; *p* = 0.002). Negative correlations were also found in Cz (*r* = -0.36; *p* = 0.009), FCz (*r* = -0.36; *p* = 0.011), and TL (*r* = -0.35; *p* = 0.012), but they did not survive correction for multiple correlations.

#### Alpha and age

Significance level for correlations was corrected to *p* < 0.009. In FXS participants, alpha did not correlate with age in any ROI. In ASD participants, positive correlations were found across almost all ROIs (Cz: *r* = 0.35, *p* = 0.016; FR: *r* = 0.33, *p* = 0.023; FL: *r* = 0.32, *p* = 0.029; FCz: *r* = 0.37, *p* = 0.01; Oz: *r* = 0.38, *p* = 0.009; Pz: *r* = 0.36, *p* = 0.014; TR: *r* = 0.34, *p* = 0.022) except TL (*r* = 0.21; *p* = 0.16), but they did not survive correction for multiple correlations. In NT controls, alpha correlated positively with age in Cz (*r* = 0.46, *p* < 0.001), FR (*r* = 0.39, *p* = 0.006), FCz (*r* = 0.53, *p* < 0.001), Oz (*r* = 0.43, *p* = 0.002), Pz (*r* = 0.52, *p* < 0.001), and TL (*r* = 0.53, *p* < 0.001). Positive correlations were also found in FL (*r* = 0.36; *p* = 0.011) and TR (*r* = 0.35; *p* = 0.014), but they did not survive correction.

#### *Beta* and age

Significance level for correlations was corrected to *p* < 0.014. In FXS participants, beta correlated positively with age in FCz (*r* = 0.39; *p* = 0.008), Pz (*r* = 0.37; *p* = 0.011), TL (*r* = 0.48; *p* = 0.001), and TR (*r* = 0.46; *p* = 0.002). In ASD participants, positive correlations with age were found in Cz, (*r* = 0.42; *p* = 0.003), FCz (*r* = 0.53; *p* < 0.001), Oz (*r* = 0.45; *p* = 0.001), and TL (*r* = 0.51; *p* < 0.001). Positive correlations were also found in Pz (*r* = 0.35; *p* = 0.014) and TR (*r* = 0.33; *p* = 0.023), but they did not survive correction. In NT controls, beta correlated positively with age in Cz (*r* = 0.36; *p* = 0.009), FCz (*r* = 0.43; *p* = 0.002), TL (*r* = 0.45; *p* < 0.001), and TR (*r* = 0.47; *p* < 0.001). A positive correlation was also found in Pz (*r* = 0.29; *p* = 0.04), but it did not survive correction for multiple correlations.

#### Low gamma and age

Significance level for correlations was corrected to *p* < 0.012. In FXS participants, low gamma only correlated positively with age in Pz (*r* = 0.39; *p* = 0.007). Positive correlations were also found in FCz (*r* = 0.31; *p* = 0.027) and TL (*r* = 0.34; *p* = 0.021), but they did not survive correction. In ASD participants, low gamma did not correlate with age in any ROI, although positive correlations were found in Cz (*r* = 0.31; *p* = 0.033) and TL (*r* = 0.30; *p* = 0.04), but did not survive correction. In NT controls, a positive correlation with age was found in TL (*r* = 0.36; *p* = 0.010). Positive correlations were also found in FL (*r* = 0.35; *p* = 0.012) and TR (*r* = 0.35; *p* = 0.013), but they did not survive correction.

#### High gamma and age

Significance level for correlations was corrected to *p* < 0.012. In FXS participants, positive correlations with age were found across almost all ROIs (Cz: *r* = 0.50, *p* < 0.001; FL: *r* = 0.41, *p* = 0.006; FCz: *r* = 0.58, *p* < 0.001; Oz: *r* = 0.55, *p* < 0.001; Pz: *r* = 0.56, *p* < 0.001; TL: *r* = 0.42, *p* = 0.005; TR: *r* = 0.44, *p* = 0.002) except FR (*r* = 0.36; *p* = 0.017), which did not survive correction. However, in ASD participants, high gamma did not correlate with age in any ROI. In NT controls, high gamma only correlated positively with age in FL (*r* = 0.36; *p* = 0.010).

### Resting state markers

#### Signal complexity

**Fig. 2:**
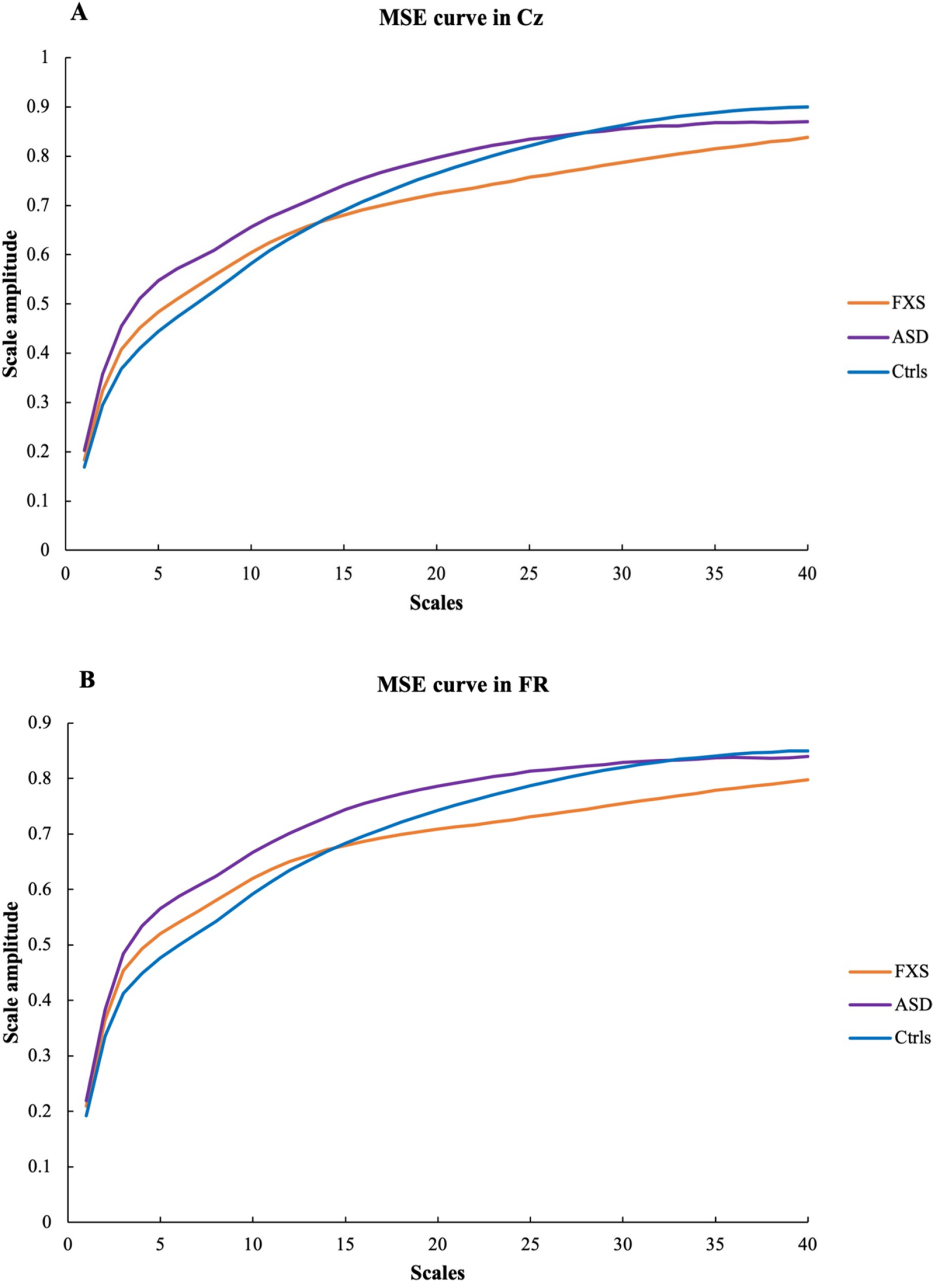
MSE curve in Cz (**A**) and FR (**B**) for FXS (orange), ASD (purple), and NT controls (blue)

Figure 2 shows the MSE curve in the central (A) and frontal right (B) regions for the three groups.

#### Lower and higher complexity scales

Results showed a significant Scales*Group interaction (*F*_(2,135)_ = 14.47, *p* < 0.001, *η*^*2*^ = 0.18), suggesting that values in the lower and higher scales differed between the three groups. Further analyses were performed to identify group differences in lower and higher complexity scales.

#### Lower complexity scales (S1-20)

MANCOVA with age as covariate showed significant effects of Age (*F*_(8,128)_ = 13.03, *p* < 0.001, *η*^*2*^ = 0.45) and Group (*F*_(16,256)_ = 4.29, *p* < 0.001, *η*^*2*^ = 0.21). Bonferroni-corrected post hoc analyses revealed reduced complexity in lower scales in NT controls compared to ASD participants in all ROIs (Cz: *p* = 0.002; FL: *p* = 0.019; FR: *p* = 0.004; FCz: *p* = 0.002; Oz: *p* = 0.005; Pz: *p* = 0.005; TL: *p* = 0.002; TR: *p* = 0.014), and diminished complexity in lower scales in FXS participants compared to ASD participants in Cz (*p* = 0.009), FCz (*p* = 0.009), Oz (*p* = 0.031), Pz (*p* = 0.043), and TL (*p* = 0.025). No differences were found between FXS participants and NT controls. MANCOVA with age and NVIQ as covariates showed significant effects of Age (*F*_(8,127)_ = 12.10, *p* < 0.001, *η*^*2*^ = 0.43) and Group (*F*_(16,254)_ = 4.18, *p* < 0.001, *η*^*2*^ = 0.21), but no significant effect of NVIQ (*F*_(8,127)_ = 1.64, *p* = 0.12, *η*^*2*^ = 0.09). Post hoc analyses with Bonferroni corrections showed decreased complexity in lower scales in NT controls compared to ASD participants in all ROIs (Cz: *p* = 0.003; FL: *p* = 0.013; FR: *p* = 0.005; FCz: *p* < 0.002; Oz: *p* = 0.008; Pz: *p* = 0.008; TL: *p* = 0.003; and TR: *p* = 0.017) when controlling for NVIQ. However, no differences were found between FXS and ASD participants, and again, there were no differences between FXS participants and NT controls.

#### Higher complexity scales (S21-40)

Results showed a significant effect of Group (*F*_(2,126)_ = 6.15, *p* = 0.003, *η*^*2*^ = 0.09). Bonferroni-corrected follow-up ANOVA revealed reduced complexity in higher scales in FXS participants compared to NT controls in all ROIs (Cz: *p* = 0.002; FL: *p* = 0.025; FR: *p* = 0.024; FCz: *p* = 0.005; Oz: *p* < 0.001; Pz: *p* < 0.001; TL: *p* = 0.015; TR: *p* < 0.001), and compared to ASD participants in all ROIs (Cz: *p* = 0.005; FL: *p* = 0.020; FR: *p* = 0.004; FCz: *p* = 0.042; Oz: *p* = 0.019; Pz: *p* = 0.004; TR: *p* = 0.018) except TL (*p* = 0.07). No differences were found between ASD participants and NT controls. Results with NVIQ as covariate showed no effects of NVIQ (*F*_(1,125)_ = 0.00, *p* = 0.99, *η*^*2*^ = 0.00) or Group (*F*_(2,125)_ = 2.19, *p* = 0.12, *η*^*2*^ = 0.03), and no differences between the groups were found in any ROI.

#### Power spectral density

**Fig. 3:**
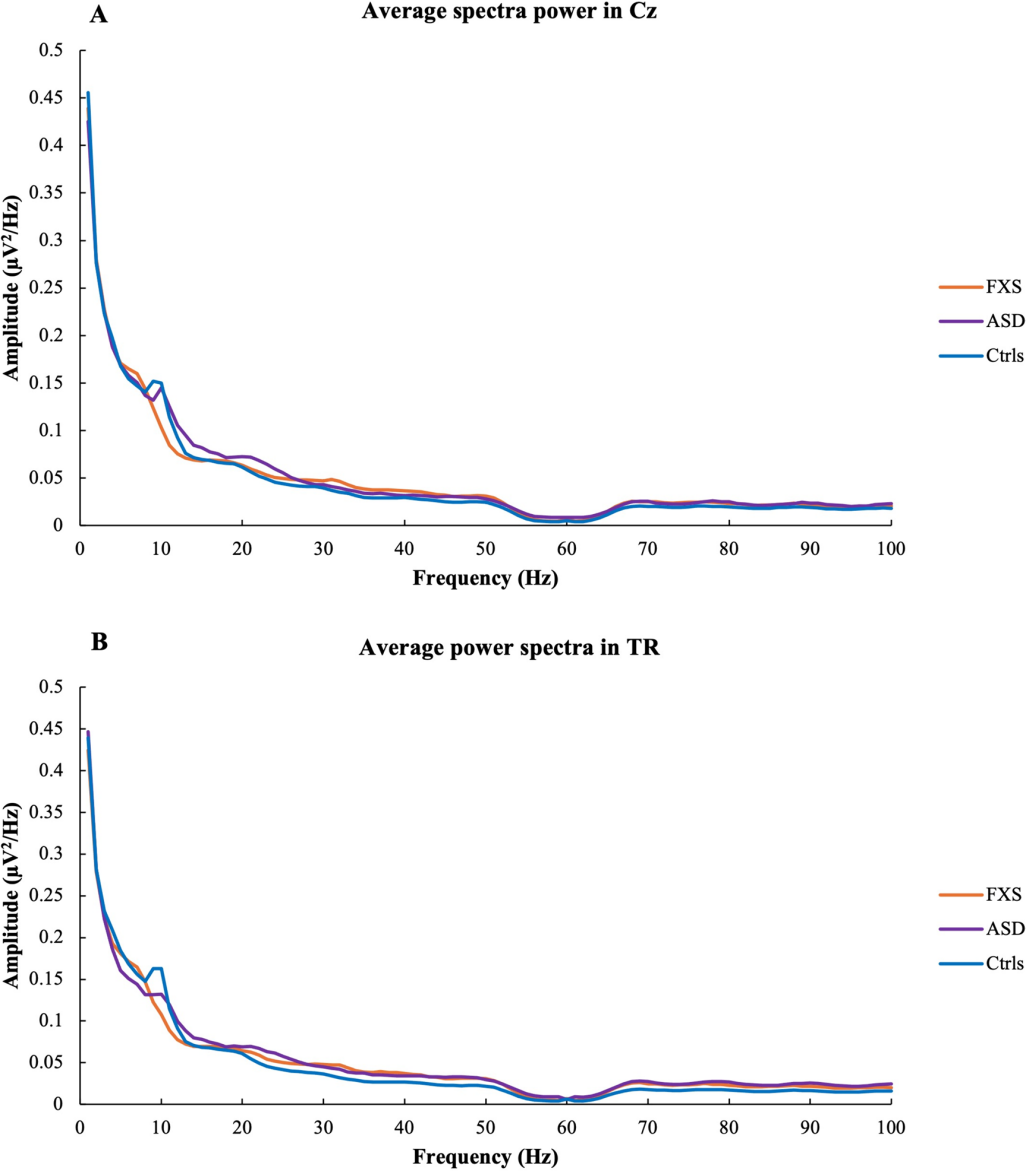
Group average spectra in Cz (**A**) and TR (**B**) for FXS (orange), ASD (purple), and NT controls (blue)

Figure 3 shows the average power spectra in the central (A) and temporal right regions (B) for the three groups.

##### APF

**Fig. 4:**
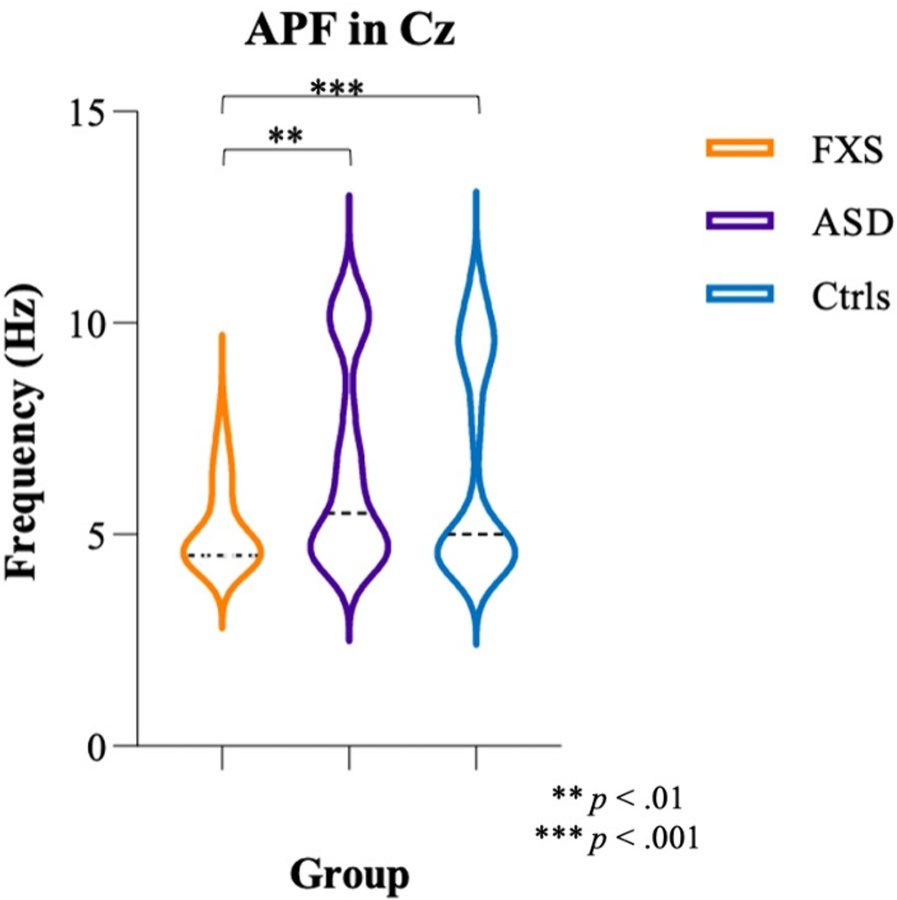
APF in Cz between FXS (orange), ASD (purple), and controls (blue). Dotted lines indicate the median for each group

MANCOVA with age as covariate showed significant effects of Age (*F*_(8,130)_ = 2.60, *p* = 0.012, *η*^*2*^ = 0.14) and Group (*F*_(16,260)_ = 1.80, *p* = 0.031, *η*^*2*^ = 0.10). Bonferroni-corrected post hoc analyses revealed lower APF in FXS participants compared to NT controls in all ROIs (Cz: *p* < 0.001; FL: *p* = 0.002; FR: *p* < 0.001; FCz: *p* < 0.001; Oz: *p* = 0.005; Pz: *p* = 0.002; TL: *p* < 0.001; TR: *p* = 0.001), as well as lower APF in FXS compared to ASD participants in Cz (*p* = 0.002), FL (*p* = 0.048), FR (*p* = 0.01), FCz (*p* = 0.005), Pz (*p* = 0.031), and TR (*p* = 0.035). No differences were found between ASD participants and NT controls. Figure 4 shows differences in APF in Cz between the three groups. MANCOVA with age and NVIQ as covariates only revealed a main effect of Age (*F*_(8,129)_ = 3.01, *p* = 0.004, *η*^*2*^ = 0.16). No NVIQ (*F*_(8,129)_ = 1.11, *p* = 0.36, *η*^*2*^ = 0.06) or Group (*F*_(16,258)_ = 0.82, *p* = 0.66, *η*^*2*^ = 0.05) effects were observed, and no differences between the groups were found in any ROI.

#### Delta

MANCOVA with age as covariate showed a significant effect of Age (*F*_(8,121)_ = 5.82, *p* < 0.001, *η*^*2*^ = 0.27), but no effect of Group (*F*_(16,242)_ = 0.65, *p* = 0.83, *η*^*2*^ = 0.04). No differences were found between the groups in any ROI. MANCOVA with age and NVIQ as covariates showed a significant effect of Age (*F*_(8,120)_ = 5.05, *p* < 0.001, *η*^*2*^ = 0.25), but no effects of NVIQ (*F*_(8,120)_ = 0.59, *p* = 0.78, *η*^*2*^ = 0.04) or Group (*F*_(16,240)_ = 0.60, *p* = 0.87, *η*^*2*^ = 0.04) were observed. Again, no differences were found between the groups in any ROI.

#### Theta

MANCOVA with age as covariate showed a significant effect of Group (*F*_(16,136)_ = 4.31, *p* < 0.001, *η*^*2*^ = 0.33), but no effect of Age (*F*_(8,118)_ = 1.61, *p* = 0.13, *η*^*2*^ = 0.10). Follow-up analyses with Bonferroni corrections revealed higher theta power in FXS compared to NT controls in all ROIs (Cz: *p* = 0.012; FL: *p* = 0.001; FR: *p* < 0.001; FCz: *p* = 0.007; Oz: *p* = 0.027; Pz: *p* < 0.001; TL: *p* = 0.041) except TR (*p* = 1.00), and compared to ASD participants in FR (*p* = 0.004), FCz (*p* < 0.001), Pz (*p* = 0.015), TL (*p* < 0.001), and TR (*p* = 0.005). NT controls showed higher theta power compared to ASD participants in TL (*p* = 0.009) and TR (*p* = 0.006), but these differences did not reach the threshold. MANCOVA with age and NVIQ as covariates showed a significant effect of Group (*F*_(16,234)_ = 3.01, *p* < 0.001, *η*^*2*^ = 0.17), but no effects of Age (*F*_(8,117)_ = 1.56, *p* = 0.14, *η*^*2*^ = 0.10) or NVIQ (*F*_(8,117)_ = 0.63, *p* = 0.75, *η*^*2*^ = 0.04) were observed. Bonferroni-corrected follow-up analyses showed increased theta power in FXS participants compared to ASD participants in FCz (*p* = 0.002) and TL (*p* = 0.003), as well as higher theta power in NT controls compared to ASD participants in TL (*p* = 0.004) and TR (*p* = 0.002), but these differences did not reach 25% of scalp coverage.

#### Alpha

MANCOVA with age as covariate showed significant effects of Age (*F*_(8,121)_ = 2.96, *p* = 0.005, *η*^*2*^ = 0.16) and Group (*F*_(16,242)_ = 3.90, *p* < 0.001, *η*^*2*^ = 0.21). Follow-up analyses with Bonferroni corrections revealed lower alpha power in FXS participants compared to NT controls in all ROIs (Cz: *p* < 0.001; FL: *p* < 0.001; FR: *p* < 0.001; FCz: *p* < 0.001; Oz: *p* < 0.001; Pz: *p* = 0.003; TL: *p* < 0.001; TR: *p* < 0.001). FXS participants also showed lower alpha power compared to ASD participants in Cz (*p* = 0.012), FL (*p* = 0.001), FR (*p* = 0.01), Oz (*p* < 0.001), Pz (*p* = 0.004), and TR (*p* = 0.032). ASD participants showed reduced alpha power compared to NT controls in TL (*p* = 0.022) and TR (*p* = 0.001), but these differences did not reach the threshold. MANCOVA with age and NVIQ as covariates showed significant effects of Age (*F*_(8,122)_ = 2.98, *p* = 0.004, *η*^*2*^ = 0.17) and Group (*F*_(16,240)_ = 2.76, *p* < 0.001, *η*^*2*^ = 0.16), but no effect of NVIQ (*F*_(8,120)_ = 0.76, *p* = 0.64, *η*^*2*^ = 0.05) was found. Bonferroni-corrected follow-up analyses showed reduced alpha power in FXS participants compared to NT controls in TL (*p* = 0.004) and TR (*p* < 0.001), and compared to ASD participants in FL (*p* = 0.033) and Oz (*p* = 0.015). Lower alpha power was also observed in ASD participants compared to NT controls in TL (*p* = 0.038) and TR (*p* = 0.004). However, none of these differences reached the threshold of 25% of scalp coverage.

#### Beta

MANCOVA with age as covariate showed significant effects of Age (*F*_(8,117)_ = 6.93, *p* < 0.001, *η*^*2*^ = 0.32) and Group (*F*_(16,234)_ = 2.32, *p* = 0.003, *η*^*2*^ = 0.14). Follow-up analyses with Bonferroni corrections revealed higher beta power in ASD participants compared to NT controls in all ROIs (Cz: *p* = 0.002; FL: *p* < 0.001; FR: *p* < 0.001; FCz: *p* < 0.001; Oz: *p* = 0.003; Pz: *p* < 0.001; TL: *p* = 0.003; TR: *p* < 0.001). Beta power was also higher in ASD participants compared to FXS participants in FL (*p* = 0.002), FR (*p* = 0.015), and Oz (*p* = 0.024). No differences were found between FXS participants and NT controls. MANCOVA with age and NVIQ as covariates showed significant effects of Age (*F*_(8,116)_ = 5.97, *p* < 0.001, *η*^*2*^ = 0.29) and Group (*F*_(16,232)_ = 1.81, *p* = 0.031, *η*^*2*^ = 0.11), but no effect of NVIQ (*F*_(8,116)_ = 0.89, *p* = 0.52, *η*^*2*^ = 0.06) was observed. Follow-up analyses with Bonferroni corrections showed higher beta power in ASD participants compared to NT controls in all ROIs (Cz: *p* = 0.004; FL: *p* < 0.001; FR: *p* < 0.001; FCz: *p* < 0.001; Oz: *p* = 0.002; Pz: *p* < 0.001; TL: *p* = 0.008; TR: *p* < 0.001) when controlling for NVIQ. No differences were found between FXS participants and NT controls, or between FXS and ASD participants.

#### Low gamma

**Fig. 5:**
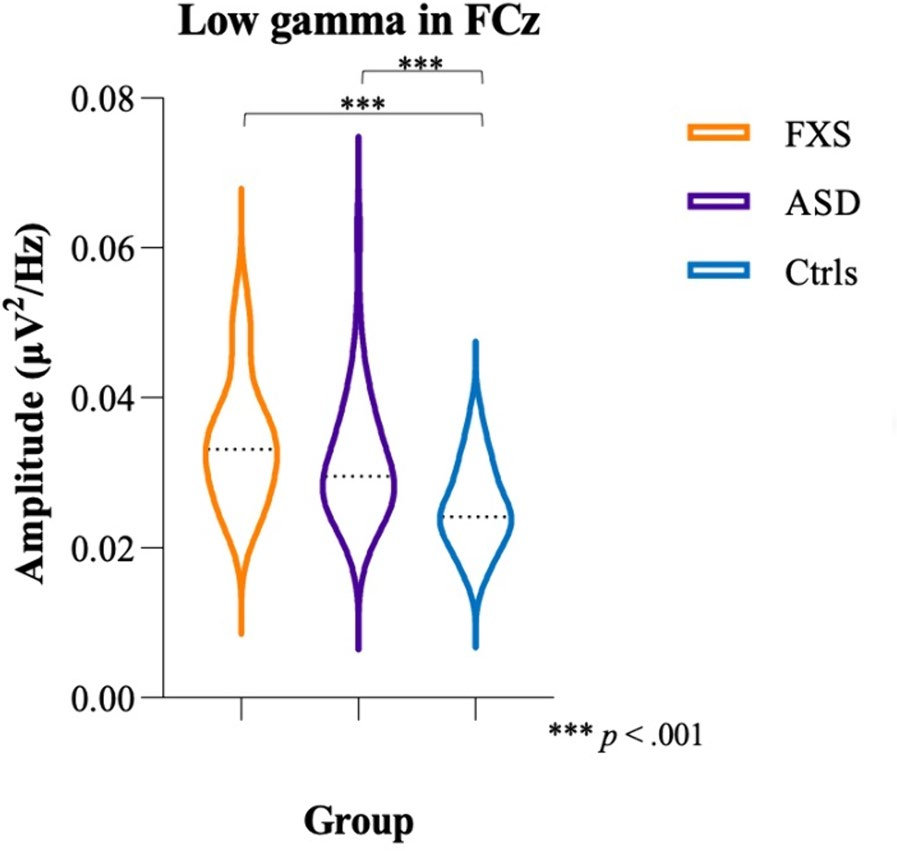
Low gamma power in FCz between FXS (orange), ASD (purple), and NT controls (blue). Dotted lines indicate the median for each group

MANCOVA with age as covariate showed significant effects of Age (*F*_(8,118)_ = 2.49, *p* = 0.016, *η*^*2*^ = 0.14) and Group (*F*_(16,236)_ = 4.20, *p* < 0.001, *η*^*2*^ = 0.22). Bonferroni-corrected post hoc analyses revealed exaggerated low gamma power in FXS participants compared to NT controls in Cz (*p* < 0.001), FR (*p* = 0.03), FCz (*p* < 0.001), Pz (*p* = 0.031), TL (*p* < 0.001), and TR (*p* < 0.001), as well as higher low gamma power in ASD participants compared to NT controls in all ROIs (Cz: *p* = 0.014; FL: *p* = 0.009; FR: *p* = 0.031; FCz: *p* < 0.001; Pz: *p* = 0.019; TL: *p* < 0.001; TR: *p* < 0.001) except Oz (*p* = 1.00). No differences were found between FXS and ASD participants. Figure 5 shows differences in low gamma power in FCz between the three groups. MANCOVA with age and NVIQ as covariates showed main effects of Age (*F*_(8,117)_ = 2.36, *p* = 0.021, *η*^*2*^ = 0.14) and Group (*F*_(16,234)_ = 2.94, *p* < 0.001, *η*^*2*^ = 0.17), but no effect of NVIQ (*F*_(8,117)_ = 0.13, *p* = 1.00, *η*^*2*^ = 0.01) was found. Post hoc analyses with Bonferroni corrections showed exaggerated low gamma power in FXS participants compared to NT controls in Cz (*p* = 0.006), FCz (*p* < 0.001), TL (*p* = 0.005), and TR (*p* = 0.002) when controlling for NVIQ. Increased low gamma power was also observed in ASD participants compared to NT controls in all ROIs (Cz: *p* = 0.012; FL: *p* = 0.007; FR: *p* = 0.33; FCz: *p* < 0.001; Pz: *p* = 0.018; TL: *p* < 0.001; TR: *p* < 0.001) except Oz (*p* = 1.00). Again, no differences were found between FXS and ASD participants.

#### High gamma

MANCOVA with age as covariate showed a significant effect of Group (*F*_(16,240)_ = 3.72, *p* < 0.001, *η*^*2*^ = 0.20), but no effect of Age (*F*_(8,120)_ = 1.52, *p* = 0.16, *η*^*2*^ = 0.09). Bonferroni-corrected post hoc analyses showed exaggerated high gamma power in ASD participants compared to NT controls in Cz (*p* = 0.009), FL (*p* = 0.043), FCz (*p* = 0.001), TL (*p* < 0.001), and TR (*p* < 0.001). Increased high gamma power was observed in ASD participants compared to FXS participants in FL (*p* = 0.016) and TL (*p* = 0.017), but these differences did not reach the threshold. No differences were found between FXS participants and NT controls. MANCOVA with age and NVIQ as covariates only showed a main effect of Group (*F*_(16,238)_ = 3.09, *p* < 0.001, *η*^*2*^ = 0.17), but no effects of Age (*F*_(8,119)_ = 1.28, *p* = 0.26, *η*^*2*^ = 0.08) or NVIQ (*F*_(8,119)_ = 0.24, *p* = 0.98, *η*^*2*^ = 0.02) were found. Bonferroni-corrected post hoc analyses showed exaggerated high gamma power in ASD participants compared to NT controls in Cz (*p* = 0.024), FCz (*p* = 0.007), TL (*p* < 0.001), and TR (*p* < 0.001) when controlling for NVIQ. High gamma power was also higher in ASD participants compared to FXS participants in FL (*p* = 0.045) and TL (*p* = 0.018), but it did not reach the 25% of scalp coverage. Again, no differences were found between FXS participants and NT controls.

## Discussion

Our study suggests both shared and unshared electrophysiological alterations in children and adolescents with FXS and ASD. Both groups showed increased low gamma power compared to NT controls; this effect remained when controlling for NVIQ, suggesting that low gamma is a powerful biomarker in both conditions. ASD participants showed exaggerated high gamma power compared to NT controls, as well as increased complexity in lower scales compared to participants with FXS and NT controls. Participants with FXS contrasted with ASD participants and NT controls by showing reduced complexity in higher scales, lower APF and alpha power, and increased theta power. Notably, however, when controlling for NVIQ, most complexity and APF differences disappeared. Our results support previous reports suggesting that signal complexity and APF may be influenced by cognitive functioning in addition to brain maturation [27, 29].

### Signal complexity

We conducted MSE analyses to assess signal complexity, revealing regularity versus stochasticity in the signal over several scales. Figure 1 illustrates MSE values. Distinctive progression of complexity with increasing scales is observed between the clinical populations. Progression stagnates around scale 15 for the FXS cohort. In contrast, the ASD cohort shows a more pronounced augmentation up to scale 20 before coming to a halt. When quantified into lower scales (pertaining to fine-grained signal) and higher scales (pertaining to coarser signal) we find that complexity in lower scales differs significantly in ASD participants, while complexity in higher scales differs significantly in FXS participants. Specifically, ASD participants showed increased complexity in lower scales, while FXS participants showed decreased complexity in higher scales. Hence, our ASD group of participants showed greater stochasticity in fine grained scales, while our FXS participants showed greater regularity in coarser scales. Atypical signal complexity in the ASD population has been highlighted in several studies [24, 25, 39]. However, these studies revealed somewhat contradictory results, a common occurrence in ASD literature due to the heterogeneity of this population. Supporting our findings, Takahashi and colleagues [40] and Ghanbari and colleagues [41] found enhanced complexity values in lower scales when measured with magnetoencephalography (MEG). Similarly, Ghanbari and colleagues [41] found brain region- and spectral band-specific patterns of complexity alterations, reflecting atypical neural dynamics in ASD. While increased complexity in lower scales and increased gamma power could share the same underlying mechanisms of excitability, our FXS participants did not exhibit the same complexity alterations in lower scales despite showing increased low gamma power. More recently, Hadoush and colleagues [42] suggested that the severity of ASD symptoms could contribute to signal complexity. They found that participants with mild ASD had significantly greater signal complexity than participants with severe ASD [42]. In the present study, all ASD participants were verbal and manifested fewer behavioral and adaptative functioning symptoms than FXS participants. As participants with FXS manifested larger developmental delays and altered cognitive functioning compared to participants with ASD, these findings could reflect cognitive functioning. Hence, variables underlying symptomatic characteristics appear to impact the mechanisms behind altered brain signal complexity in clinical populations, exemplified by the absence of alterations observed in FXS when controlling for NVIQ.

An increase in signal complexity is generally expected with age in NT populations [29]. Our study revealed an age-related increase in lower complexity scales in NT controls as well as in ASD and FXS participants, suggesting an evolution of signal complexity in lower scales during development in both clinical populations. These findings further confirm our previous findings in FXS [20]. In contrast, we did not find age-related changes in higher scales in either of the groups. Recent evidence of increased complexity in finer scales but decreased complexity in coarser scales from childhood to adolescence [43] has been interpreted as a reduction of adaptability in long-range connections during adolescence. Our study may not have sufficient power to capture nonlinear developmental changes during adolescence. Nevertheless, FXS participants exhibited decreased complexity in higher scales, which can be interpreted as reduced adaptability of long-range connections. This interpretation is further supported by the fact that a significant portion of the variance in these results is attributable to cognitive functioning levels. Disentangling these findings in larger samples will be necessary for more definitive conclusions.

### APF

Alpha peak frequency is a well-known EEG marker for brain maturation and is thought to increase with age [44], shifting from the theta range to the alpha range during normal development. Here, no age effects were observed in FXS, but NT controls showed a typical increase with age. Furthermore, the present study is among the first to explore age-related changes in APF in ASD participants and we revealed an age-related increase in APF in our sample of participants with a NVIQ predominantly in the normal range.

APF was significantly lower in FXS participants compared to ASD participants and NT controls, but no differences were found between ASD participants and NT controls. Although literature on APF in FXS is scarce, our findings are consistent with previous results showing reduced APF in FXS [20, 45]. Moreover, the absence of APF differences in our ASD group could be related to their normal level of cognitive functioning. One previous study highlighted the potential effect of both age and cognitive levels on APF in ASD participants [27]. Interestingly, no significant differences were observed between the groups when controlling for NVIQ. The considerably lower NVIQ scores in most FXS participants compared to the other groups make it challenging to disentangle the effects of the developmental condition from the level of cognitive functioning. Nevertheless, our findings provide new insight into the brain maturation of verbal participants with ASD manifesting mild cognitive and behavioral symptoms. Literature on APF in adults with FXS and ASD is lacking, and more research investigating this marker will be needed to gain further knowledge on the intricacies of APF and its relation to age in neurodevelopmental conditions.

### PSD

Individuals with ASD are generally observed to present a U-shaped pattern of altered spectral power: exaggerated power in low frequency (delta and theta) and high frequency (beta and gamma) bands but reduced power in the middle-range frequency (alpha) band [46]. Similar patterns of altered PSD have been reported in FXS [20, 21]. Unexpectedly, our PSD results showed no differences in delta power between groups, even when controlling for NVIQ, although excessive delta power has been reported in both FXS and ASD [18, 20]. Our results are consistent with another study reporting no differences in delta power between FXS participants and controls [21]. However, in ASD, results for delta power seem to conflict. Shephard and colleagues [47] observed lower delta power in participants with comorbid ASD and ADHD compared to controls and lower delta power in participants with only ADHD compared to participants with only ASD. These results indicate that ADHD may influence delta power, suggesting that the high prevalence of comorbid ADHD in our FXS and ASD participants could partially explain the absence of group differences in delta power. More specifically, the presence of ADHD could attenuate delta power in our clinical populations. Other studies have reported inconsistent results in delta power between low- and high-functioning children with ASD [46]. Both reduced and augmented delta power have been reported in children with high-functioning ASD compared to NT children [48, 49], but another study reported lower delta power in both low- and high-functioning children with ASD [50]. Evidence of a reduction in delta activity during development in NT subjects has been reported repeatedly [29, 47]. Interestingly, our correlation analyses showed a decrease in delta power with age in all three groups, suggesting that this trend is also present in neurodevelopmental conditions.

In the present study, FXS participants showed elevated theta and reduced alpha power compared to NT controls, supporting previous literature [20, 21, 51]. In fact, lower APF and increased theta power may be related to the same underlying phenomenon in our FXS group. Our results also showed higher theta power in most ROIs in FXS participants compared to ASD participants. In contrast, ASD participants showed no significant differences in theta and alpha power. Once again, because NVIQ contributed significantly to this pattern, the absence of differences in the ASD group may be due to their higher level of functioning. Increased theta power has been reported in low-functioning children with ASD [52], but not high-functioning [50]. While evidence of reduced alpha power has been consistent in low-functioning children with ASD [46], diverging results have been reported in high-functioning children with ASD, who show enhanced alpha power compared to NT children [53, 54]. These inconsistencies suggest that the heterogeneity of ASD has a crucial impact on theta and alpha rhythms. Similar to delta activity, theta power is known to decrease during normal development [47, 55]. Here, we observed an age-related decrease in theta power in NT controls and FXS participants, but no association with age was found in ASD participants.

Notably, no differences in beta power were found between FXS participants and NT controls. These results are consistent with previous literature showing either no difference in beta power between FXS and NT controls [21], or decreased low beta (13-20 Hz) power in FXS individuals [45]. However, beta power was higher in ASD participants compared to NT controls in all ROIs, and the same results were observed when controlling for NVIQ. These results align with previous evidence of elevated beta power in ASD [48, 56, 57], suggesting that elevated beta power is a strong biomarker. Individuals with mutations in the SYNGAP1 gene, which are strongly associated with ASD and ID, also showed higher beta power during an auditory task [58, 59]. Our results also suggest that elevated beta power is a strong resting state biomarker in ASD regardless of cognitive functioning. Beta power was also higher in ASD participants compared to FXS participants in frontal and occipito-central regions. Given that elevated beta power has been reported widely in ASD, these results are not surprising. The literature has revealed an increase in fast wave activity during childhood in NT children [55]. Here, an age-related increase in beta power was found in both clinical groups, as well as in NT controls. Our results suggest that these typical maturational changes also occur in FXS and ASD, although a notable excess in beta power was observed in ASD participants. Whether this distinct pattern of brain rhythms in resting state activity reflects a more specific phenotype in ASD will need further investigation.

Low gamma power was significantly and similarly higher in FXS and ASD participants compared to NT controls. These results were expected, as they confirm robust evidence of perturbated low gamma oscillations in both individuals with FXS [20, 21] and ASD [22, 46, 53, 57]. Moreover, the same patterns of low gamma power were observed when controlling for NVIQ, supporting the conclusion that low gamma power is a powerful resting state biomarker in neurodevelopmental conditions regardless of the severity of cognitive impairments. Furthermore, high gamma power was enhanced in ASD participants, but not FXS, compared to NT controls in most ROIs. Although high gamma activity has not been as widely investigated as low gamma, these results are consistent with previous studies showing elevated high gamma power in participants with ASD compared to NT controls [53]. The same results were observed when controlling for NVIQ. However, the insufficient amount of literature investigating resting state EEG oscillations of greater than 70 Hz in FXS and ASD is concerning. Further studies are required to better understand how higher frequencies are altered in these neurodevelopmental conditions, as well as the impact of cognitive functioning on these oscillations. Evidence of age-related increases in high frequencies during neurodevelopment has been reported [55]. In the current study, low and high gamma were positively associated with age in FXS participants and NT controls in some ROIs, supporting previous literature. However, gamma did not correlate with age in ASD participants in any ROI, suggesting that gamma power might already be elevated at a young age in this population.

### Specific alterations in FXS and ASD

Delayed brain maturation and hyperexcitability are two important mechanisms involved in resting state EEG alterations. More specifically, elevated theta power and reduced APF and signal complexity are widely recognized as brain maturation markers [20, 27, 29, 60, 61], whereas reduced alpha power and enhanced beta and gamma power have been associated with hyperexcitability in neurodevelopmental disorders [20, 21, 57]. Our results emphasize the evidence of delayed and abnormal brain maturation in FXS. Indeed, FXS participants showed reduced signal complexity and APF, as well as higher theta power compared to both NT controls and ASD participants. These markers are thought to be associated with synaptic abnormalities. The extensive research focusing on synaptic development in FXS has shown that loss of FMRP expression results in dendritic spine malformations and synaptic overgrowth, directly affecting neurodevelopment [18, 62]. Thus, in addition to contributing to the EEG alterations in FXS, these neurodevelopmental functional impairments could explain the cognitive stagnation reported in early childhood in FXS and contribute to the severe behavioral symptoms observed in this population. The current study also supports the notion of hyperexcitability in FXS. We observed reduced alpha power and exaggerated low gamma power. In FXS individuals with the full mutation, the absence of FMRP leads to dysregulation of mRNA translation, causing excessive protein synthesis and altered synaptic plasticity (Bassel & Warren, 2008). Hence, impairments in excitatory (glutamate) and inhibitory (GABA) neurotransmission are reported in FXS, reflected by deficits in the GABAergic system and resulting in increased gamma power [21, 62]. GABAergic inhibitory interneurons underlie EEG alpha oscillations, which modulate external sensory processing [62, 63]. Therefore, decreased alpha power is thought to be induced by a failure to inhibit insignificant sensory information in FXS [51]. Overall, our results support the evidence of hyperactive glutamatergic and hypoactive GABAergic mechanisms in FXS and offer a better understanding of how this imbalance is reflected in resting state EEG. We also observed elevated beta and gamma power in our ASD participants compared to NT controls. These results support the hyperexcitability theory in our ASD cohort, as abnormalities in the inhibitory system have been reported in ASD as well [18]. This reduction in GABAergic activity has been linked to decreased alpha power and increased power in higher frequencies [46]. Hence, our findings of altered gamma power are consistent with the literature and suggest hyperactive cortical mechanisms in both populations.

## Conclusions

Our study confirms that several resting state EEG markers are altered in FXS and ASD participants. Given that our ASD participants manifested fewer cognitive and behavioral impairments compared to our FXS participants, direct comparison of the two conditions was difficult, and caution is required in interpreting the results. Replicating this study with more impaired individuals with ASD is imperative in order to provide a better understanding of the variables affecting these alterations. Nevertheless, the similarities between the two conditions remain powerful demonstrations of atypical EEG activity in these populations. Overall, our results indicate that low gamma power is a robust biomarker in both neurodevelopmental conditions despite significant cognitive functioning differences. The question of whether exaggerated density of high frequency activity relates phenotypically to levels of impairment in other cognitive domains or biologically to underlying mechanisms of excitability remains to be addressed more specifically in later work. Our findings strongly suggest that the main EEG alterations observed in FXS and ASD could serve as powerful biomarkers of treatment response in future clinical studies.

## Data Availability

All data are available in this manuscript. Further inquiries can be directed to the corresponding author.

## Abbreviations

ABC-C-FX: Aberrant Behavior Checklist for Community, FX version
ADHD: Attention deficit hyperactivity disorder
ADOS-2: Autism Diagnostic Observation Schedule, Second Edition
APF: Alpha peak frequency
ASD: Autism spectrum disorder
CBD: Cannabidiol
CGG: Cytosine-guanine-guanine
DSM-5: Diagnostic and Statistical Manual of Mental Disorders, Fifth Edition
EEG: Electroencephalogram
FFT: Fast Fourier transform
FMR1: Fragile X messenger ribonucleic 1
FXS: Fragile X syndrome
ID: Intellectual disability
MEG: Magnetoencephalography
mGluRs: Metabotropic glutamate receptors
MSE: Multiscale entropy
NT: Neurotypical
NVIQ: Nonverbal intellectual quotient
PSD: Power spectral density
ROIs: Regions of interest
SB-5: Stanford-Binet Intelligence Scales, Fifth Edition
Vineland-III: Vineland Adaptative Behavior Scales, Third Edition
WASI- II: Wechsler Abbreviated Scale of Intelligence, Second Edition
